# Differences in the Responses of Photosystems I and II in *Cymbidium sinense* and *C. tracyanum* to Long-Term Chilling Stress

**DOI:** 10.3389/fpls.2015.01097

**Published:** 2016-01-05

**Authors:** Jia-Wei Li, Shi-Bao Zhang

**Affiliations:** ^1^Key Laboratory for Economic Plants and Biotechnology, Kunming Institute of Botany, Chinese Academy of SciencesKunming, China; ^2^Yunnan Key Laboratory for Wild Plant ResourcesKunming, China; ^3^University of Chinese Academy of SciencesBeijing, China

**Keywords:** cyclic electron flow long-term chilling stress, orchid, photoinhibition, photoprotection

## Abstract

The susceptibility of photosystem I (PSI) and photosystem II (PSII) to chilling stress depends on plant species, and cyclic electron flow (CEF) plays an important role in photoprotection for some species under short stress periods. However, little is known about the responses of PSI and PSII to long-term chilling stress. We studied two orchid species—*Cymbidium sinense* and *C. tracyanum—* that differ in their capacity to adapt to low temperature, and exposed plants for 19 d to stress conditions that included 4°C and a light intensity of 250 to 350 μmol photons m^-2^ s^-1^. Meanwhile, we investigated their dynamic variations in Chl fluorescence and P700 parameters. After exposure to 4°C and 250 μmol photons m^-2^ s^-1^ for 6 h, PSI activity was maintained stable in both species, but stronger PSII photoinhibition was observed in *C. sinense.* During the long-term treatment, the maximum quantum yield of PSII was significantly reduced, with that decrease being greater in *C. sinense.* After 19 d of chilling treatment, the maximum photo-oxidizable P700 declined only slightly in *C. tracyanum* but dropped significantly in *C. sinense.* Linear electron flow was largely depressed during the long-term chilling treatment, especially in *C. sinense.* Meanwhile, *C. tracyanum* showed higher CEF activity than *C. sinense.* These results indicate that PSII is more sensitive to chilling-light stress than PSI in both species. The rate of PSII photodamage at chilling-light stress is higher in *C. sinense* than *C. tracyanum*, and CEF contributes to photoprotection for PSI and PSII under long-term chilling stress in *C. tracyanum.*

## Introduction

Members of the genus *Cymbidium* are well-known horticultural plants. However, most of its species are seriously endangered due to human activities (especially over-collection), destruction of natural habitats, and climate change (Luo et al., 2003; Liu et al., 2009). Although awareness and efforts to conserve these valuable resources are gradually increasing, little is known about the photosynthetic mechanisms by which species within this genus adapt to environmental stresses such as long-term chilling.

*Cymbidium* plants are widely distributed in tropical and subtropical Asia, and also in northern Australia, where enormous habitat diversification has occurred (Yukawa et al., 2002; Zhang et al., 2002). This genus has three life forms—terrestrial, epiphytic, and lithophytic (Motomura et al., 2008). Among them, the epiphytic species benefit from exposure to higher light intensities and have relatively little competition, but must sometimes survive with limited supplies of water and nutrients (Zotz and Tyree, 1996; Laube and Zotz, 2003; Zotz and Schmidt, 2006; Zotz, 2013; Zotz and Winkler, 2013). *Cymbidium sinense* in particular is very sensitive to environmental changes: a combination of low relative humidity and drought can reduce photosynthesis rate and the accumulation of nutrients, inhibit floral bud differentiation and diminish population survival (Liu et al., 2009). Kuang and Zhang (2015) have reported that, under strong irradiance, the activities of photosystem II (PSII) and photosystem I (PSI) are more drastically decreased in *C. sinense* than in *C. tracyanum*, and they have suggested that activation of cyclic electron flow around PSI (CEF) in *C. tracyanum* under high light is a primary photoprotective mechanism.

Naturally, leaves of *C. tracyanum* undergo long-term low temperature stress in winter compared with *C. sinense.* Exposure to low temperatures can cause photoinhibition in chilling-sensitive plants, such as cucumber *(Cucumis sativus*, Sonoike, 1999) and tropical tree species (Huang et al., 2010a). Photoinhibition is a phenomenon in which photosynthetic efficiency declines because the input of photons exceeds photosynthetic requirements (Powles, 1984). In cucumber, both PSII and PSI activities are significantly reduced after treatment for 5 h at 4°C and 200 μmol photons m^-2^ s^-1^ (Sonoike, 1995, 1999). For tropical trees, PSII is more sensitive than PSI to chilling stress associated with moderate intense light (Huang et al., 2010a). After exposure to 4°C and 150 μmol photons m^-2^ s^-1^ for 8 h, PSI activity significantly decreased in *Arabidopsis thaliana* (Zhang and Scheller, 2004). These previous studies have spanned only a few hours. However, little is known about how long-term chilling stress influences photosynthetic performance in *Cymbidium* species.

Several protective mechanisms contribute to the prevention of photoinhibition, such as CEF and non-photochemical quenching (NPQ; Munekage et al., 2002, 2004; Takahashi et al., 2009; Tikkanen et al., 2014), those responses can affect how plants adapt to excess light energy. For example, CEF is an important mechanism by which PSI is protected from photoinhibition under high light in *Arabidopsis thaliana* (Munekage et al., 2002; Kono et al., 2014; Tikkanen et al., 2014). This may result because CEF not only can directly oxidize P700 into P700^+^, but can also consume excess reducing-power NADPH (Shikanai, 2007). The CEF-generated proton gradient across the thylakoid membrane (ΔpH) enables the repair of photo-damaged PSII and also suppresses photodamage to PSII (Takahashi et al., 2009). However, the function of CEF in protecting PSII and PSI under chilling stress remains controversial (Sonoike, 1998; Thomas et al., 1999; Ivanov et al., 2001; Kim et al., 2001; Barth and Krause, 2002). Kim et al. (2001) have reported that stimulation of CEF protects PSI from further photodamage in cucumber plants chilled in the light. By contrast, Barth and Krause (2002) have indicated that NDH-pathway CEF does not protect PSI in tobacco leaves illuminated at a chilling temperature. It is unknown whether the CEF contributed to photoprotection during long-term chilling treatment.

The study presented here takes an approach that integrates chlorophyll fluorescence and the P700 redox state. In particular, we examined the effect of chilling stress on PSI and PSII in *Cymbidium sinense* and *C. tracyanum*, two species with contrasting life forms and temperature sensitivities. We addressed the following questions: (1) Do these species show differential responses by PSII and PSI during long-term chilling, and (2) Is CEF relatively stimulated under such stress to alleviate photoinhibition? As part of our examination, we specifically tested the hypothesis that *C. tracyanum* is less vulnerable to chilling-light stress than *C. sinense* because plants of the latter species are naturally distributed at lower elevations, thus, are not as well-adapted to long-term low temperature.

## Materials and Methods

### Plant Materials

*Cymbidium sinense* is a typical terrestrial orchid species always found on forest floors or in well-drained, shaded thickets at elevations from 300 to 1500 m. By contrast, the epiphytic *C. tracyanum* occurs on tree trunks in forests or on rocks along streams at 1200 to 2000 m. To minimize the potential effect of developmental differences on our experimental results, we selected mature individuals of fairly uniform size, and propagated research materials via tissue culture. The new plants were placed in plastic pots containing a bark mixture and grown in a greenhouse (20% full sunlight, 17 to 27°C, and 50 to 70% relative humidity) at the Kunming Institute of Botany, Kunming, China.

### Photoinhibitory Treatments

To determine the response of PSI and PSII to short-term chilling stress, detached leaves incubated in the presence or absence of lincomycin (1 mM) overnight in darkness were transferred to 4°C and 300 μmol photons m^-2^ s^-1^ for 6 h. The long-term chilling treatment was conducted in two phytotrons in which temperature, humidity, light intensity, and CO_2_ concentration were controlled. Ten healthy plants per species were used. The temperature was maintained at 20 to 27°C (natural growth temperature; control) in one phytotron and at 4 ± 1°C (chilling temperature) in the other. For leaves of both species, light intensity was controlled at 250 to 350 μmol photons m^-2^ s^-1^ for 10 h each day. Other experimental conditions included 60 to 80% relative humidity (vapor pressure deficit of 0.53 to 1.12 kPa) and a CO_2_ concentration of 400 μmol mol^-1^. Chlorophyll fluorescence and P700 parameters were recorded on Days 1, 5, 12, 15, and 19 of the treatment period.

### Chlorophyll Fluorescence Measurement

The *in vivo* chlorophyll fluorescence of PSII was measured with a Dual PAM-100 (Heinz Walz, Effeltrich, Germany) connected to a computer with control software. Minimum fluorescence *(F_o_*) and maximum fluorescence (*F_m_*) were determined before four dark-adapted leaves from individual plants of each species were exposed to artificial light. Those leaves were then used for deriving light-response curves after they were light-adapted (240 μmol photons m^-2^ s^-1^) for at least 15 min. Light-adapted fluorescence parameters were also evaluated for the same leaves after 18 min of exposure to increasingly stronger photosynthetic photon flux densities (PPFDs) that began at 37 μmol photons m^-2^ s^-1^ and then increased stepwise to 61, 94, 150, 190, 297, 363, 555, and finally 684 μmol photons m^-2^ s^-1^.

The following chlorophyll fluorescence parameters were calculated: the maximum quantum yield of PSII after dark-adaptation overnight, *F_v_/F_m_ = (F_m_ - F_o_*)/*F_m_*; the minimum fluorescence in the light-adapted state, *F_o_′ = F_o_/(F_v_/F_m_ + F_o_/F_m_′)* (Oxborough and Baker, 1997); coefficient of photochemical quenching, qP = (*F_m_*′ -*F_s_)/(F_m_′ - F_o_′);* effective quantum yield of PSII, Y(II) = (*F_m_′ - F_s_)/F_m_′* (Genty et al., 1989); NPQ = (*F_m_ - F_m_′)/F_m_′* (Baker, 2008); fraction of energy dissipated in the form of heat via the regulated, Y(NPQ) = *F_s_/F_m_′- F_s_/F_m_* (Kramer et al., 2004); and fraction of energy passively dissipated in the form of heat and fluorescence, Y(NO) = *F_s_/F_m_* (Hendrickson et al., 2004; Kramer et al., 2004). Here, *F_v_* represented variable fluorescence; *F_m_*, dark-adapted maximum fluorescence; *F_o_*, dark-adapted minimum fluorescence; *F_m_′*, light-adapted maximum fluorescence; and *F_s_*, light-adapted steady state fluorescence.

### P700 Measurement

The P700 redox state was measured by a Dual PAM-100 with a dual wavelength unit (830/875 nm), following the method of Klughammer and Schreiber (1994). Saturation pulses (10,000 μmol photons m^-2^ s^-1^) were used. Maximum photo-oxidizable P700 *(P_m_)* was determined by applying a saturation pulse after pre-illumination with far-red light (Huang et al., 2010a,b; Suorsa et al., 2012; Tikkanen et al., 2014). The P700^+^ signals (*P*) may vary between a minimal (P700 fully reduced) and a maximal level (P700 fully oxidized). As a defined optical property, the amplitude of *P_m_* depends upon the maximum amount of photo-oxidizable P700, which represents the quantity of efficient PSI complex. The maximum change in P700 in a given light state (*P_m_*′) is measured when actinic light is used instead of far-red illumination. The photochemical quantum yield of PSI [Y(I)], calculated as *(P_m_′ - P)/P_m_*, is defined as the fraction of overall P700 that is reduced and not limited by the acceptor side in a given state. Parameter [Y(ND)] is estimated as *P*/*P_m_* and represents the fraction of overall P700 that is oxidized in a given state that is enhanced by a *trans*-thylakoid proton gradient and photodamage to PSII. In addition, Y(NA) represents the fraction of overall P700 that cannot be oxidized by a saturation pulse in a given state due to a lack of acceptors, which was calculated as *(P_m_ - P_m_*′*)/P_m_.*

### Estimation of Photosynthetic Electron Flow

Photosynthetic electron flow through PSI and PSII was calculated as follows: ETRI = Y(I) × PPFD × 0.84 × 0.5, ETRII = Y(II) × PPFD × 0.84 × 0.5, where 0.84 represents the leaf absorbance and 0.5 is the proportion of absorbed light energy allocated to PSI or PSII. Finally, we estimated cyclic electron flow around PSI, or CEF, as the difference in electron flow between PSI and PSII (Miyake et al., 2005; Huang et al., 2012, 2015).

### Statistical Analysis

Statistical analysis was performed with SPSS 16.0. Data were subjected to analysis of variance (ANOVA), and Tukey’s multiple comparison tests were used to determine whether significant differences existed between treatments at α = 0.05.

## Results

### Responses of PSI and PSII to Short-Term Chilling Treatment

**FIGURE 1:**
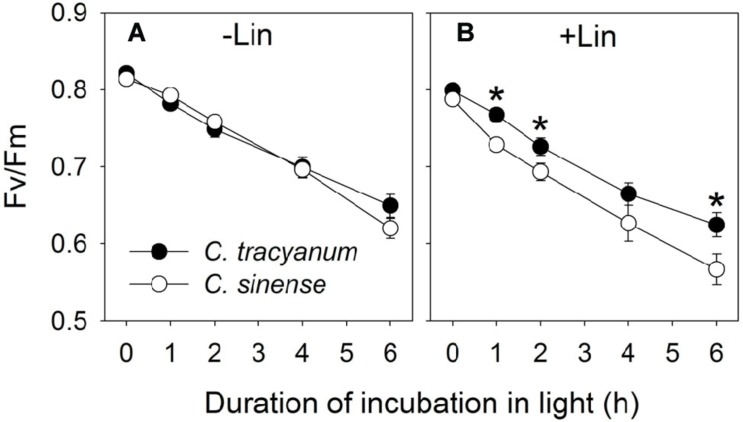
**Change in the maximum quantum yield of PSII after dark adaptation (*F_v_*/*F_m_*) in *Cymbidium sinense* and *C. tracyanum* during chilling treatment at 4°C and 250 μmol photons m^-2^ s^-1^ in the presence (B) and absence (A) of lincomycin**. The mean ± SE was calculated from at least 5 plants.

**FIGURE 2:**
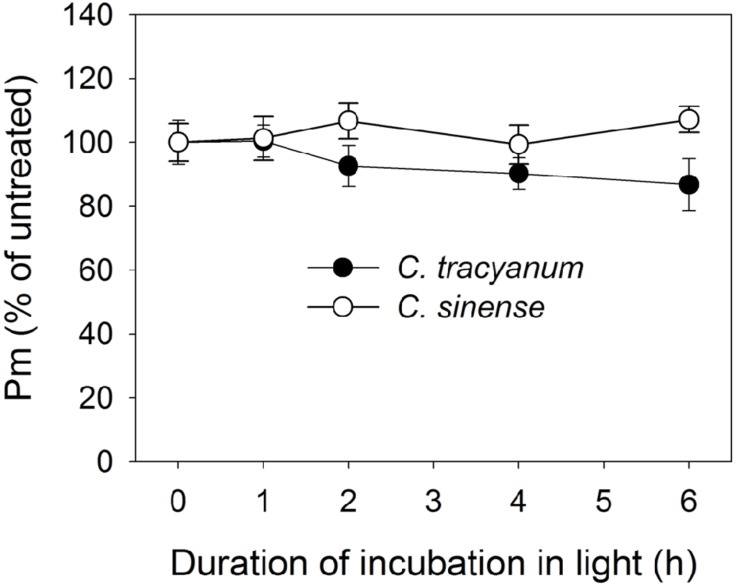
**Change in the maximum photo-oxidizable P700 (*P_m_*) in *Cymbidium sinense* and *C. tracyanum* during chilling treatment at 4°C and 250 μmol photons m^-2^ s^-1^**. The mean ± SE was calculated from at least 5 plants.

In order to determine whether the rate of PSII photodamage at chilling-light stress differs between the two species, detached leaves were pre-incubated with lincomycin. Lincomycin, an inhibitor of chloroplast translation machinery, prevents the *de novo* synthesis of D1 protein and thus stops the turnover of PSII. The presence of lincomycin made it possible to evaluate the rate of PSII photodamage. During chilling treatment at 4°C and 300 μmol photons m^-2^ s^-1^ without lincomycin, both species showed the same extent of decrease in *F_v_/F_m_* (**Figure 1A**). However, with pre-treatment by lincomycin, *C. sinense* showed larger decrease in *F_v_/F_m_* than *C. tracyanum* (**Figure 1B**), indicating that the rate of PSII photodamage at this chilling-light stress was greater in *C. sinense.* After 6 h treatment, *P_m_* did not significantly differ compared to the original value in both species (**Figure 2**), suggesting that PSI was insusceptible to short-term chilling-light stress in both species.

### Response of PSII to Long-Term Chilling Treatment

**FIGURE 3:**
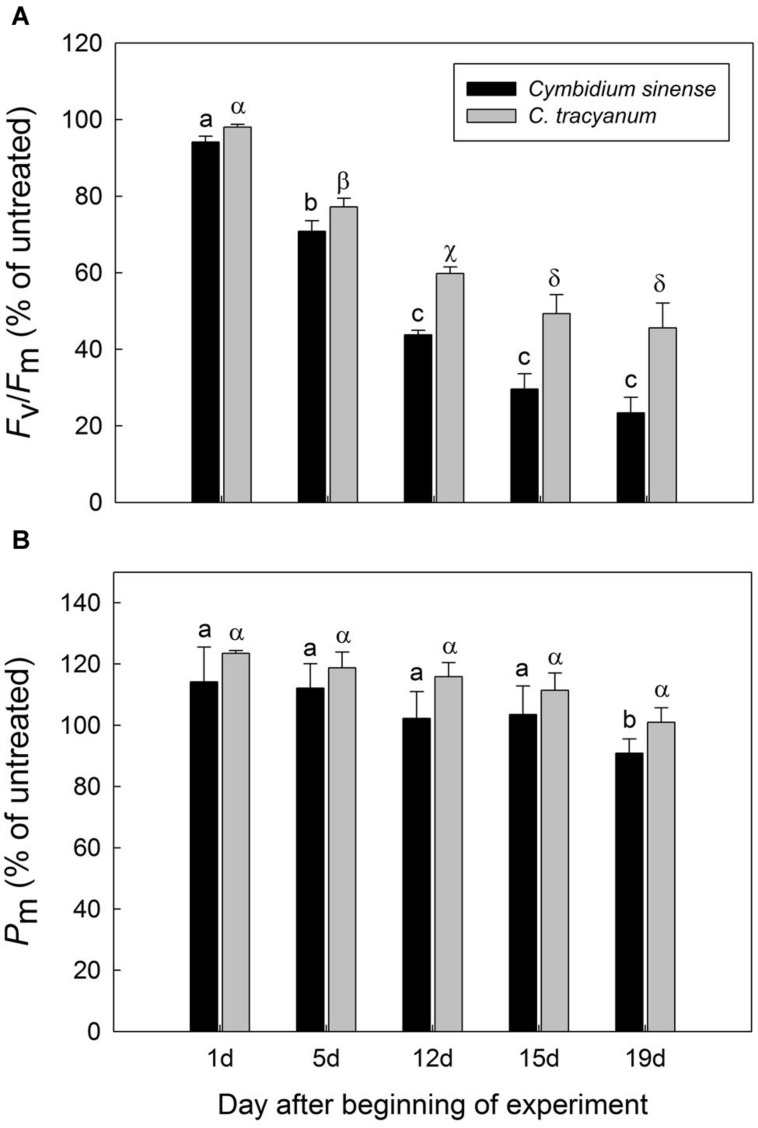
**Variations in the maximum quantum yield of PSII after dark adaptation (*F_v_*/*F_m_*) (A) and maximum photo-oxidizable P700 (*P_m_*) (B) for *Cymbidium sinense* and *C. tracyanum* plants exposed to chilling stress (4°C)**. Each vertical bar represents mean ± SE for 4 measurements from individual plants. Different letters above bars indicate significant differences in *F_v_/F_m_* or *P_m_* between treatments (*P* < 0.05, based on ANOVA, followed by Tukey’s *post hoc* tests for comparison).

**FIGURE 4:**
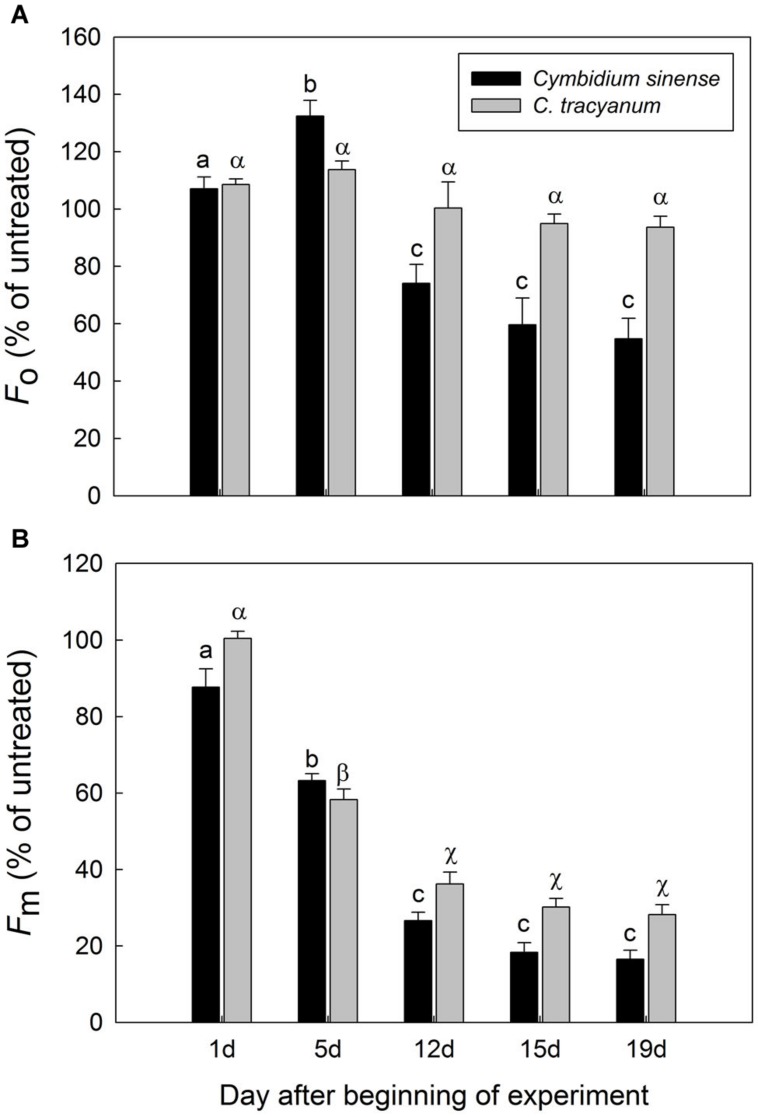
**Changes in *F_o_* (A) and *F_m_* (B) of *Cymbidium sinense* and *C. tracyanum* plants during chilling-stress treatment (4°C)**. Each vertical bar represents mean ± SE for four measurements from individual plants. Different letters above bars indicate significant differences in *F_o_* or *F_m_* between treatments (*P* < 0.05, based on ANOVA, followed by Tukey’s *post hoc* tests for comparison).

After 5 d of exposure to chilling stress, the maximum quantum yield of PSII after dark-adaptation *(F_v_/F_m_)* in both species was significantly reduced, with the amplitude of this decrease being larger in *Cymbidium sinense.* By the end of the long-term treatment, *F_v_/F_m_* had declined by 54.9% in *C. tracyanum* versus 77.8% in *C. sinense* (**Figure 3A**). During the long-term chilling treatment, both *F_o_* and *F_m_* decreased significantly in *C. sinense.* By comparison, *F_o_* changed only slightly while *F_m_* dropped significantly in *C. tracyanum* (**Figure 4**).

**FIGURE 5:**
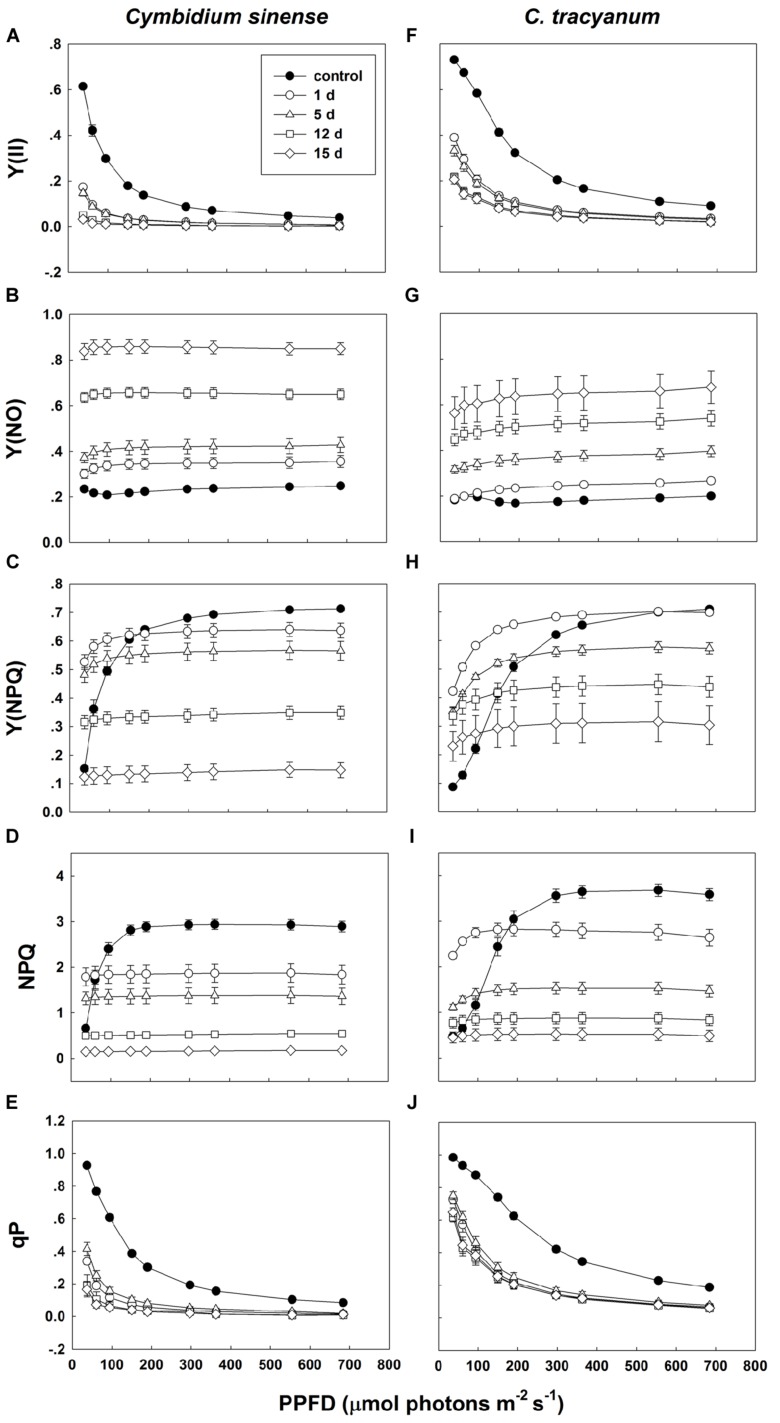
**Light-response curves of effective quantum yield of photosystem II [Y(ll)] (A,F); quantum yield of non-regulated energy dissipation [Y(NO)] (B,G); quantum yield of regulated energy dissipation [Y(NPQ)] (C,H); non-photochemical quenching (NPQ) (D,l); and photochemical quenching (qP) (E,J) in *Cymbidium sinense* and *C. tracyanum* during period of chilling stress (4°C)**. Each data point represents mean ± SE for at least 4 measurements from individual plants.

**FIGURE 6:**
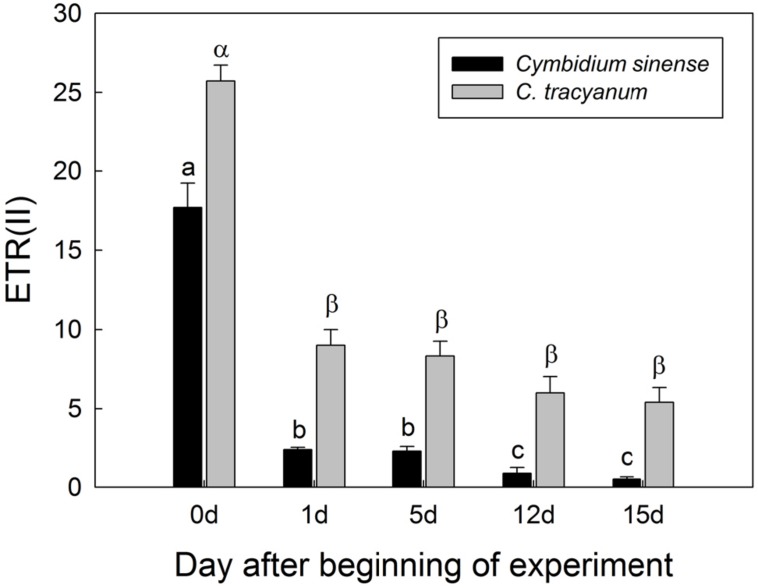
**Changes in electron transfer via PSII [ETR(II)] at 290 μmol photons m**^-^**^2^ s**^-^**^1^ in *Cymbidium sinense* and *C. tracyanum* during period of chilling stress (4°C)**. Each vertical bar represents mean ± SE for at least 4 measurements from individual plants. Different letters above bars indicate significant differences between treatments (*P* < 0.05, based on ANOVA, followed by Tukey’s *post hoc* tests for comparison).

Compared with plant status prior to treatment, values for effective quantum yield of photosystem II [Y(II)] and photochemical quenching (qP) during the chilling period significantly decreased in both species regardless of PPFD level. As the light intensity increased, Y(II) and qP decreased more rapidly in *C. sinense* (**Figures 5A,E,F,J**). The electron transfer via PSII [ETR(II)] in both species was significantly reduced after exposure to chilling stress, with the value of ETR(II) being larger in *C. tracyanum* (**Figure 6**). The quantum yield of non-regulated energy dissipation [Y(NO)] gradually increased during the experimental period; values rose more rapidly for *C. sinense* (**Figures 5B,G**). After 1 d of chilling stress, Y(NPQ) largely increased at low PPFDs in both species (**Figures 5C,H**). As the treatment time was extended, the maximum value of Y(NPQ) gradually decreased for both species. Prior to the induction of stress, the light-response curve showed that NPQ peaked at 190 μmol photons m^-2^ s^-1^ for *C. sinense* (value of 2.9) versus 360 μmol photons m^-2^ s^-1^ for *C. tracyanum* (value of 3.6) (**Figures 5D,I**). After 15 d, values for NPQ and Y(NPQ) were close to zero in *C. sinense.* Over time, both parameters showed more rapid declines in *C. sinense* than in *C. tracyanum.*

### Response of PSI to Long-Term Chilling Treatment

The maximum photo-oxidizable P700 *(P_m_)* in *C. sinense* was not significantly affected by chilling stress in the first 15 d, but did show a significant decrease after 19 d of treatment. For *C. tracyanum, P_m_* did not change significantly throughout the whole experimental period (**Figure 3B**). These results indicated that PSI was relatively insusceptible to long-term chilling-light stress compared to PSII in both species.

**FIGURE 7:**
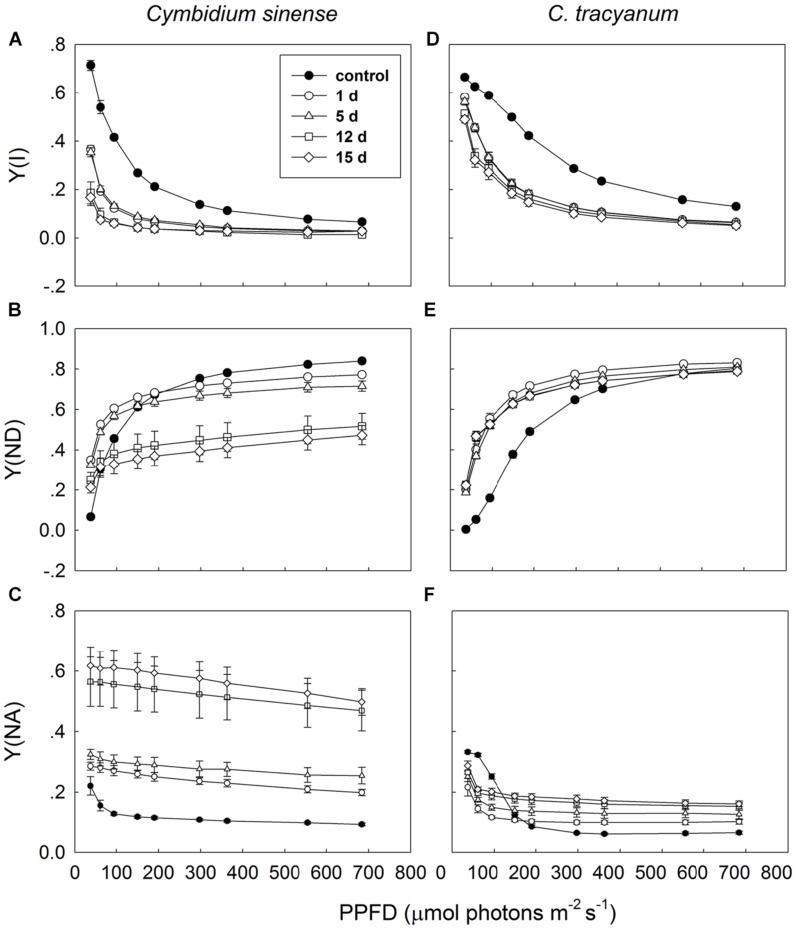
**Light-response curves of effective quantum yield of photosystem I [Y(I)] (A,D); PSI donor side limitation [Y(ND)] (B,E); and PSI acceptor side limitation [Y(NA)] (C,F) in *Cymbidium sinense* and *C. tracyanum* during period of chilling stress (4°C)**. Each data point represents mean ± SE for at least 4 measurements from individual plants.

Light-response curves indicated that, when plants were exposed to 4°C, the effective quantum yield of PSI [Y(I)] in both species rapidly decreased as the treatment was prolonged (**Figures 7A,D**). Over time, the two species displayed different trends in changes in PSI donor side limitations [Y(ND)]. Compared with pretreatment status, the value of Y(ND) under high light gradually declined with the extension of chilling treatment in *C. sinense* (**Figure 7B**). For *C. tracyanum*, Y(ND) was significantly increased at PPFDs below 190 μmol photons m^-2^ s^-1^ but was changed only slightly when plants were exposed to stronger light levels (**Figure 7E**). After 15 d of treatment, Y(NA) under high light rose to 0.5 for C. *sinense* versus 0.2 for *C. tracyanum* (**Figures 7C,F**). These results indicated that the PSI redox states for the two species responded differently to long-term chilling stress.

### Response of CEF Around PSI to Long-Term Chilling Treatment

**FIGURE 8:**
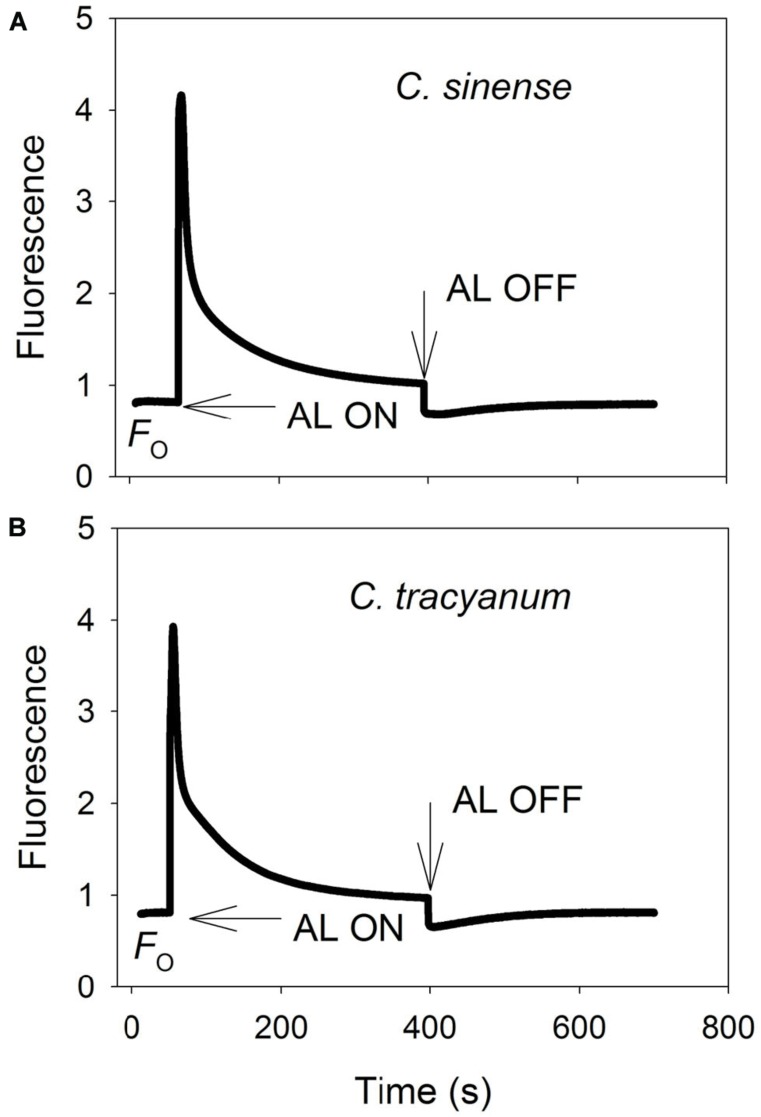
**Monitoring of NDH activity by chlorophyll fluorescence**. Mature leaves of *Cymbidium sinense* **(A)** and *C. tracyanum* **(B)** were exposed to actinic light (AL, 209 μmol photons m^-2^ s^-1^) after the measuring light was turned on (*F_o_*, the minimum level of chlorophyll fluorescence). The AL was turned off and the subsequent change in chlorophyll fluorescence was monitored as an indicator of NDH activity. The mature leaf was dark-adapted at 25°C for 30 min, and the chlorophyll fluorescence was measured at 25°C.

**FIGURE 9:**
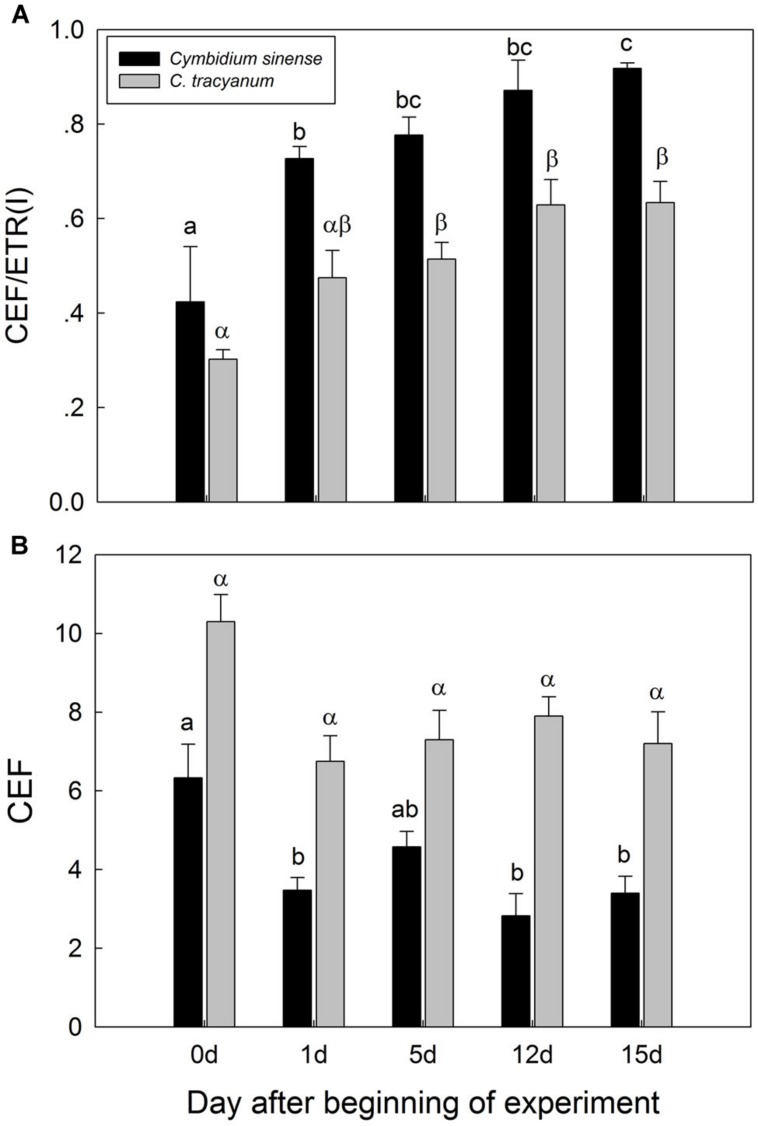
**Changes in maximum ratio of cyclic electron flow around PSI to electron transfer via PSI [CEF/ETR(I)] (A) and in cyclic electron flow around PSI (CEF) (B) at 290 μmol photons m^-2^ s^-1^ in *Cymbidium sinense* and *C. tracyanum* during period of chilling stress (4°C)**. Each vertical bar represents mean ± SE for 4 measurements from individual plants. Different letters above bars indicate significant differences between treatments *(P <*0.05, based on ANOVA, followed by Tukey’s *post hoc* tests for comparison).

It has been indicated that the NDH and PGR5 pathway explained most of the CEF in higher plants judging from the phenotype of double mutants (Johnson, 2011). NDH can be monitored by a transient post-illumination increase in chlorophyll fluorescence, due to NDH-dependent reduction of the plastoquinone pool in darkness (Yamori et al., 2011). In both species, the transient increase in chlorophyll fluorescence was observed, indicating the presence of NDH pathway in them (**Figure 8**). Furthermore, before chilling treatment, both species showed high values of Y(ND) under high light, indicating the presence of PGR5 pathway in them (Munekage et al., 2002, 2004; Kono et al., 2014). During the long-term chilling treatment, ETRII largely decreased in both species, and CEF decreased in less extent. As a result, the maximum ratio of CEF to ETRI, i.e., CEF/ETR(I)*_max_*, increased gradually as the treatment time was extended (**Figure 9A**). During the long-term chilling treatment, the value of CEF at 290 μmol photons m^-2^ s^-1^ was higher in *C. tracyanum* than in *C. sinense* (**Figure 9B**).

## Discussion

Chilling-light stress is a typical climate in winter in subtropical regions. However, the responses of PSI and PSII to long-term chilling-light stress is little known. In our present study, we investigated the effect of long-term chilling-light stress on PSI and PSII activities in two chilling-tolerant *Cymbidium* species C. *sinense* and *C. tracyanum.* We found that PSI was relatively insusceptible to long-term chilling-light stress than PSII in these two species. Furthermore, after 19 d of chilling treatment, *C. sinense* showed stronger PSII photoinhibition than *C. tracyanum*, which was associated with the lower CEF activity in *C. sinense.*

### PSII Activity Under Long-Term Chilling Stress

The transfer of electrons from PSII to PSI can be used by downstream electron sink pathways, including those for the processes of photosynthetic carbon fixation and photorespiration. Low environmental temperatures can decrease the rates of those processes, ultimately increasing ROS production, which then aggravates the photoinhibition of PSI (Sonoike, 2006) and PSII (Murata et al., 2007; Takahashi et al., 2009). We showed here that the maximum quantum yield of PSII after dark-adaptation was significantly reduced in both species under chilling stress. This indicated that PSII activity was sensitive to chilling-light stress in both species.

Photodamage to PSII is correlated with the oxygen-evolving complex (OEC), which is located on the luminal side of the thylakoid membrane. Once the OEC is inactivated, the PSII reaction center is then disrupted by the absorption of light (Hakala et al., 2005; Ohnishi et al., 2005). Repair of PSII, a crucial step that possibly determines the extent of PSII photodamage, requires several steps: (1) degradation of damaged D1 protein, (2) *de novo* synthesis of D1 protein, and (3) enrolling of newly synthesized D1 protein into PSII. The synthesis of D1 protein is especially inhibited by environmental stress (Allakhverdiev and Murata, 2004), which can lead to ROS production (Murata et al., 2007). Although we did not determine the status of D1 protein in this study, values for *F_v_/F_m_* and Y(NO), are considered good indicators of PSII photodamage (Takahashi et al., 2009; Huang et al., 2010b). Here, we found that *F_v_/F_m_* and Y(NO) increased gradually in both species during the chilling period, especially in *C. sinense*, thereby demonstrating that excess light energy could not be consumed through photochemical quenching and NPQ. Before chilling stress was induced, C. *sinense* had a lower NPQ value, which further suggested that this species is less able to dissipate excess energy. Moreover, the rate of PSII photodamage was significantly higher in *C. sinense* than *C. tracyanum.* These results might be partially explained by the contrast in their natural habitats. That is, *C. sinense* probably receives less light because it grows on forest floors rather than on tree trunks, as is the case with *C. tracyanum.* Therefore, the latter species would have greater ability to cope with stress associated with excess light energy (Kuang and Zhang, 2015). Furthermore, plants of *C. sinense* are generally distributed at lower elevations, which might also explain why it differs from *C. tracyanum* in the extent of its chilling-induced PSII photoinhibition.

### The Role of CEF in Photoprotection Under Long-Term Chilling Stress

Our results showed that, during the chilling period, values for CEF measured at 290 μmol photons m^-2^ s^-1^ were significantly higher in *C. tracyanum* than in *C. sinense.* This result was consistent with the difference of PSII photoinhibition between the two species. These results suggested that CEF plays an important role in alleviating PSII photoinhibition when plants of these species are subjected to chilling temperatures.

CEF-dependent development of a *trans*-thylakoid membrane proton gradient helps to alleviate photoinhibition through at least two mechanisms. One is linked to NPQ generation, which prevents PSII recovery from being inhibited while the second mechanism suppresses the rate of photodamage (Takahashi et al., 2009). Theoretically, CEF-dependent generation of DpH increases lumen Ca^2+^ concentrations in the thylakoids, thereby protects the OEC against photodamage. Impairment of CEF by mutation of pgr5 accelerated the rate of PSII photodamage under high light (Takahashi et al., 2009). We found that, at chilling temperature, a reduced level of CEF activity was associated with a higher rate of PSII photodamage in *C. sinense.* Taken together, we conclude that the difference in the extent of PSII photoinhibition between these two species was partially caused by the contrast in their CEF capacities.

### PSI Activity Under Long-Term Chilling Stress

One reliable parameter, *P_m_*, can be used to reflect the quantity of maximum photo-oxidizable P700 (Zhang and Scheller, 2004; Huang et al., 2010a,b; Suorsa et al., 2012; Tikkanen et al., 2014). When comparing with PSII, we found that PSI activity in both species was only slightly affected by chilling stress. Species-dependent sensitivity of PSI to low temperatures has been reported previously. For example, PSI activity in chilling-sensitive species, such as cucumber and *Arabidopsis thaliana*, is much more vulnerable to short-term exposure (Sonoike, 1999; Zhang and Scheller, 2004). Photoinhibition of PSI under such conditions has also been described with common bean (Sonoike, 1995) and *Zea mays* (Kingston-Smith et al., 1999). However, the extent of photoinhibition is not always greater in PSI than in PSII. For example, in tropical tree species, PSII is more sensitive to chilling/light stress than PSII (Huang et al., 2010a,b). We noted here that PSI activity remained stable after 19 d of treatment, suggesting that the relative insusceptibility of PSI to combined chilling/light stress favored the survival of both species during winter.

Hydroxyl radicals that are generated by the reaction between hydrogen peroxide and photo-reduced iron-sulfur centers are the primary means by which photoinhibition of PSI is induced (Sonoike, 1996). This outcome initially occurs at iron-sulfur centers on the acceptor side. One indicator, Y(NA), is used to represent the acceptor side limitation of PSI (Suorsa et al., 2012; Kono et al., 2014). We found that, during the treatment period, Y(NA) increased gradually in *C. sinense* but was only slightly changed in *C. tracyanum.* Consequently, each species showed different responses in their PSI redox states to chilling stress. Photoinhibitory damage of PSI can be induced by over-reducing on the PSI acceptor side (Shuvalov et al., 1986; Golbeck, 1987; Munekage et al., 2002), behavior that depends upon the active transfer of electrons from PSII to PSI. If this transfer is blocked, PSI activity remains stable under high light, as demonstrated with pgr5 mutants of *Arabidopsis thaliana* (Munekage et al., 2002; Suorsa et al., 2012; Tikkanen et al., 2014). For cucumber leaves, pre-treatment under darkness prevents photoinhibition of PSI when plants are later exposed to combined light and chilling treatment because PSII is inhibited under such conditions (Higuchi et al., 2003). Tikkanen et al. (2014) have proposed that control of PSII photoinhibition is the ultimate regulator of the electron transfer chain, and it provides a photoprotective mechanism against ROS formation and photodamage to PSI. Our results also indicated that, for both species, ETR(II) largely decreased during the chilling/light treatment. Thus, we largely attribute the relative stability of PSI to the depression of LEF and/or photodamage of PSII.

In summary, our findings demonstrate that *C. sinense* is more sensitive to long-term chilling stress than *C. tracyanum*, and that PSII is more susceptible than PSI in both species. At the lower temperature, greater CEF capacity in *C. tracyanum* alleviated chilling-induced PSII photoinhibition. During the experimental period, the depression of electron transfer from PSII to PSI and/or PSII photoinhibition prevented the photoinhibition of PSI. Therefore, our findings provide evidence that the insusceptibility of PSI to long-term chilling-light stress favors the survival of both orchid species when exposed to low temperatures in winter.

## Author Contributions

J-WL and S-BZ: Conceived and designed the experiments. J-WL and S-BZ: Performed the experiment. J-WL and S-BZ: Analyzed the data. J-WL and S-BZ: Contributed to the writing of the manuscript.

## Conflict of Interest Statement

The authors declare that the research was conducted in the absence of any commercial or financial relationships that could be construed as a potential conflict of interest.
